# AI-based organ weight estimation from postmortem computed tomography

**DOI:** 10.1038/s41598-026-62085-2

**Published:** 2026-07-14

**Authors:** Marc Windgassen, Andreas Heinrich

**Affiliations:** 1 Institute of Forensic Medicine, Jena University Hospital, Friedrich Schiller University, Am Klinikum 1, 07747 Jena, Germany; 2 Department of Radiology, Jena University Hospital, Friedrich Schiller University, Am Klinikum 1, 07747 Jena, Germany

**Keywords:** X-ray computed tomography, Body weights and measures, Body composition, Forensic medicine, Computer-assisted image processing, Artificial intelligence, MeSH, Anatomy, Diseases, Health care, Medical research

## Abstract

Accurate organ weight determination is essential in forensic autopsy. Postmortem computed tomography (CT) combined with Artificial Intelligence (AI)-based segmentation allows automated, non-invasive organ delineation. This study evaluated the accuracy of CT-derived organ weights against conventional autopsy measurements. A total of 100 postmortem CT examinations (mean age 54 ± 21 years; 35 females, 65 males) were analyzed. Brain, heart, liver, kidneys, and spleen were segmented automatically in two model resolutions, and organ weights were derived from voxel volumes and Hounsfield unit-based tissue densities. Across all organs, mean differences between CT- and autopsy-derived organ weights were − 12.69 ± 107.96 g (median − 2.95 g) for the 1.5 mm model and − 18.42 ± 116.67 g (median − 1.95 g) for the 3 mm model. Mean absolute differences were 70.76 ± 83.07 g (median 29.05 g) and 77.43 ± 90.60 g (median 30.50 g), respectively. Agreement was highest for the brain and liver, followed by the kidneys and spleen, whereas the heart showed the largest deviations. Segmentation failures were uncommon and were mainly associated with decomposition, trauma, or incomplete organ coverage. These findings indicate that AI-based segmentation enables reliable, largely non-invasive organ weight estimation in postmortem CT and may support forensic investigations and personal identification workflows based on antemortem imaging.

**Supplementary Information:**

The online version contains supplementary material available at 10.1038/s41598-026-62085-2.

## Introduction

Organ weight measurement is essential in forensic pathology because deviations from normal values can give hints on disease (e.g. cardiac hypertrophy), or intoxication (e.g. brain edema) and affect cause-of-death determinations. Conventional autopsy provides the most accurate organ weights, yet it requires opening the body, which makes the procedure time consuming, may compromise trace evidence and is sometimes restricted by cultural or legal considerations. Postmortem computed tomography (CT) has emerged as a non-invasive option, offering detailed anatomical visualization without disturbing the body^1,2^. Although a full autopsy remains the gold standard in postmortem forensic examination, there may be a constellation of factors in which it is not warranted, e.g. for cultural reasons. Recent advances in Artificial Intelligence (AI) and image segmentation techniques have enabled automated and precise delineation of individual organs, raising the potential to estimate organ weights directly from CT data. Reliable organ weight estimation via postmortem CT could enhance forensic investigations by supporting rapid assessment of abnormal findings, and especially providing quantitative data for epidemiological studies, even without performing an autopsy. While previous research^3^ has explored automatic whole-body weight estimation from CT scans, the accuracy of automatic organ-specific weight estimation from postmortem CT has not yet been systematically validated; most existing methods still rely on manual segmentations^4–8^.

This study aims to evaluate whether AI-based segmentation of postmortem CT datasets can produce organ weight estimates that closely correspond to measurements obtained from conventional autopsies, potentially providing a fast, reproducible, and non-invasive tool for forensic practice, scientific and epidemiological purposes.

## Methods

This study was approved by the institutional review board (IRB) at Jena University Hospital (registration number 2019-1505-MV) and was carried out in accordance with relevant guidelines and regulations. Due to the retrospective nature of the investigation, the need for written informed consent was waived by the IRB at Jena University Hospital.

### Cohorts and retrospective data collection

A retrospective search of the institutional Picture Archiving and Communication System (PACS) identified all whole-body postmortem CT examinations performed between June 2015 and May 2025. Inclusion required both a whole-body postmortem CT series and a corresponding forensic autopsy report that contained documented organ-weight measurements.

**Fig. 1:**
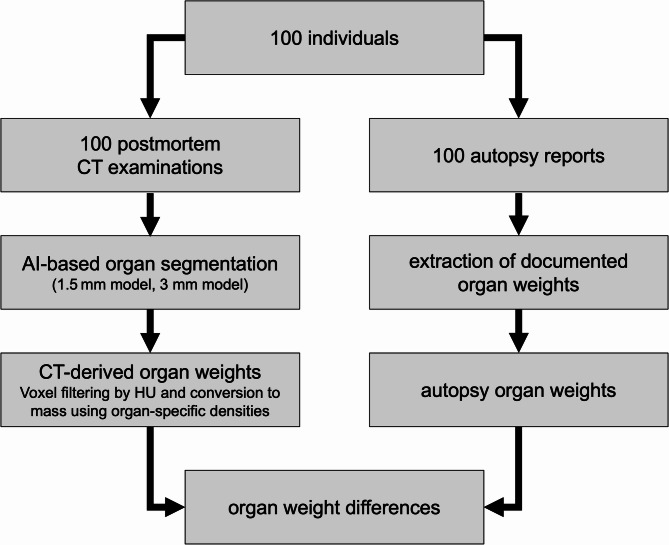
Overview of the study workflow comparing CT- and autopsy-derived organ weights in 100 individuals, including AI-based segmentation and quantitative weight estimation.

The final dataset comprised 100 postmortem CT examinations from 100 individuals (see Fig. 1). The mean age was 54 ± 21 years (median 51 years), and the cohort included 35 females and 65 males. The mean stature was 171.54 ± 10.42 cm and the mean body weight was 75.25 ± 18.62 kg. CT acquisition parameters varied across examinations and included a tube voltage of 120 kV (helical acquisition) and a tube current-time product of 222.60 ± 147.75 mAs. Slice thickness was 0.625 mm in 94 scans, 1.25 mm in 1 scan, and 2.5 mm in 5 scans. Image reconstruction was performed using a standard convolution kernel in all examinations. Iterative reconstruction (ASIR-V) was applied in 72 scans at level 50% and in 5 scans at level 60%; in 23 scans, reconstruction details were not available in the Digital Imaging and Communications in Medicine (DICOM) metadata.

Autopsy reports were examined by a board-certified forensic pathologist, who extracted the recorded weights of brain, heart, liver, spleen, left and right kidney. These organ-weight measurements served as the reference standard for comparison with the CT-derived organ weight estimates. In addition, information regarding the condition of the body, including postmortem changes, was obtained from the autopsy reports.

### Image processing

The CT datasets were first converted from DICOM to the Neuroimaging Informatics Technology Initiative (NIfTI) format. Thereafter the TotalSegmentator algorithm^9^(version 2.10.0) was run in its total mode, which simultaneously segments a predefined list of regions of interest that includes brain, liver, heart, both kidneys and spleen. To assess how processing speed affects segmentation quality, each scan was analyzed twice: first without the fast option (standard 1.5 mm resolution) and then with the fast option enabled (lower-resolution 3 mm model, which reduces memory requirements). After segmentation, only voxels assigned to each organ-specific mask were retained for further analysis. To reduce contamination from non-tissue structures, a Hounsfield unit (HU)-based filtering step was applied, retaining only voxels within a predefined attenuation range of -100 to 200 HU. The remaining voxels were counted and multiplied by voxel volume to obtain organ volumes. Organ weight was subsequently estimated using two alternative approaches. First, a fixed-density model was applied, in which organ-specific literature-based densities^10,11^ were assigned to each structure (brain: 1.04 g/cm³, heart: 1.06 g/cm³, liver: 1.06 g/cm³, kidneys: 1.05 g/cm³, spleen: 1.06 g/cm³). In this approach, organ weight was calculated as:

1 $$weight = volume \times \rho \left( {fixed} \right)$$

Second, a voxel-wise HU-dependent density model was implemented to account for spatial heterogeneity in tissue composition. In this approach, density was derived directly from CT attenuation values using a linear transformation:

2 $$\rho \left( {HU} \right) = 1 + {\text{ }}HU/1000$$

The resulting voxel-wise densities were multiplied with the corresponding voxel volumes and summed to obtain organ-specific weight estimates. The linear HU-to-density conversion is a pragmatic approximation and may not fully capture nonlinear attenuation behavior, beam-hardening effects, or postmortem gas-related artifacts. These effects may contribute to residual variability in CT-derived organ weight estimates, particularly in decomposed cases.

### Parameter optimization

To systematically evaluate the robustness of both approaches, two complementary parameter variation strategies were investigated. In the fixed-density model, the assumed density values were varied over a range from 0.80 to 1.30 g/cm³ in increments of 0.01 g/cm³. This allowed assessment of the sensitivity of organ weight estimates to deviations from literature-based reference values. In parallel, the HU-based model was scaled using multiplicative correction factors ranging from 0.80 to 1.20 (step size 0.01). This enabled evaluation of global calibration effects on the HU-derived weight estimates. For each organ and parameter configuration, absolute differences between CT- and autopsy-derived organ weights were calculated. The median absolute error was used as the primary optimization criterion instead of the mean absolute error to ensure robustness against occasional segmentation failures and extreme outliers caused by postmortem changes. Only cases fulfilling predefined organ-specific plausibility criteria were included in the analysis. Segmentation failure was defined as an umbrella term comprising complete failure of mask generation and insufficient mask quality, resulting in implausible organ weights below predefined thresholds. These plausibility thresholds were defined a priori at the beginning of the study and were not modified during parameter optimization. These thresholds excluded implausible observations when CT-derived organ weights fell below conservative limits (brain ≥ 250 g, heart ≥ 50 g, liver ≥ 200 g, each kidney ≥ 25 g, spleen ≥ 25 g). They were intentionally chosen as conservative plausibility limits to exclude only clearly implausible results caused by gross undersegmentation or failed segmentation while minimizing the risk of excluding physiologically plausible organs. In the study cohort, the predefined thresholds, which were not derived from the data, corresponded to approximately 28–50% of the lowest observed autopsy-derived organ weights across organs, illustrating their conservative nature. The best-performing density estimation approach was subsequently selected for all downstream analyses.

### Evaluation

The CT-derived organ weights were subsequently compared with the corresponding autopsy measurements. For each organ, both absolute and relative differences were computed. Analyses were performed separately for the 1.5 mm and 3 mm segmentation models, and organ weight estimation was based on a voxel-wise HU-dependent density model with organ-specific multiplicative scaling factors derived from prior parameter optimization. Segmentation failures were classified when CT-derived organ weights were missing or fell below the predefined plausibility thresholds as defined above.

In addition, the performance of CT-derived organ weight estimation was evaluated with respect to the postmortem condition of the deceased. Therefore, subgroup analyses were performed to investigate the influence of postmortem interval (PMI), decomposition, and disrupted body integrity on segmentation performance and organ weight differences. Subgroups were defined according to PMI strata (0, < 3, <5, < 10, >10 days, and unknown), decomposition status, specific pathologies (exsanguination, cerebral edema, and pulmonary edema), and the presence of major anatomical disruption resulting from polytrauma or thermal injury. All analyses were conducted separately for the 1.5 mm and 3 mm models, and only successfully segmented cases with available autopsy reference data were included.

Statistical analysis was performed using paired differences (CT minus autopsy). Differences between models (1.5 mm vs. 3 mm) were assessed using the two-sided Wilcoxon signed-rank test and Bland-Altman analysis. Bias was defined as the mean difference in CT–autopsy errors between models and limits of agreement as bias ± 1.96 standard deviations. Only cases with valid segmentations in both models were included.

## Results

Estimation of organ weights from postmortem CT data is feasible and generally shows good agreement with conventional autopsy measurements (see Table 1 and Supplementary Table S1). Across all organs, the mean difference between CT- and autopsy-derived organ weights was − 12.69 ± 107.96 g (median − 2.95 g) for the 1.5 mm model, with a mean absolute difference of 70.76 ± 83.07 g (median 29.05 g). For the 3 mm model, the mean difference was − 18.42 ± 116.67 g (median − 1.95 g), with a mean absolute difference of 77.43 ± 90.60 g (median 30.50 g).

**Table 1:** Summary statistics of CT- and autopsy-derived organ weights and their case-wise differences are reported as absolute median [Q1–Q3]. Percentage values represent the median absolute percentage deviation relative to autopsy-derived organ weights. Full descriptive statistics, including means and standard deviations, are provided in the Supplementary Table S1. Organ-specific effective sample sizes (n) used for the statistical analyses are reported in Table 2.

Parameter	Brain	Heart	Liver	Kidney (left)	Kidney (right)	Spleen
Autopsy-derived organ weights [g]	1355 ± 167	402 ± 117	1596 ± 510	150 ± 47	142 ± 43	152 ± 85
1.5 mm model CT-derived organ weights [g]	1313 ± 266	403 ± 207	1548 ± 583	147 ± 52	137 ± 52	163 ± 88
3 mm model CT-derived organ weights [g]	1305 ± 260	387 ± 198	1552 ± 554	146 ± 53	141 ± 56	180 ± 111
Differences between CT- and autopsy-derived organ weights [g]						
1.5 mm model abs. median [Q1–Q3]	44 [24, 91]	96 [65, 172]	73 [39, 172]	11 [5, 22]	11 [5, 21]	14 [5, 37]
Percentage	4	29	5	7	8	12
3 mm model abs. median [Q1–Q3]	45 [29, 90]	103 [64, 175]	70 [48, 208]	11 [6, 24]	12 [7, 25]	16 [8, 59]
Percentage	3	29	5	8	10	13

**Fig. 2:**
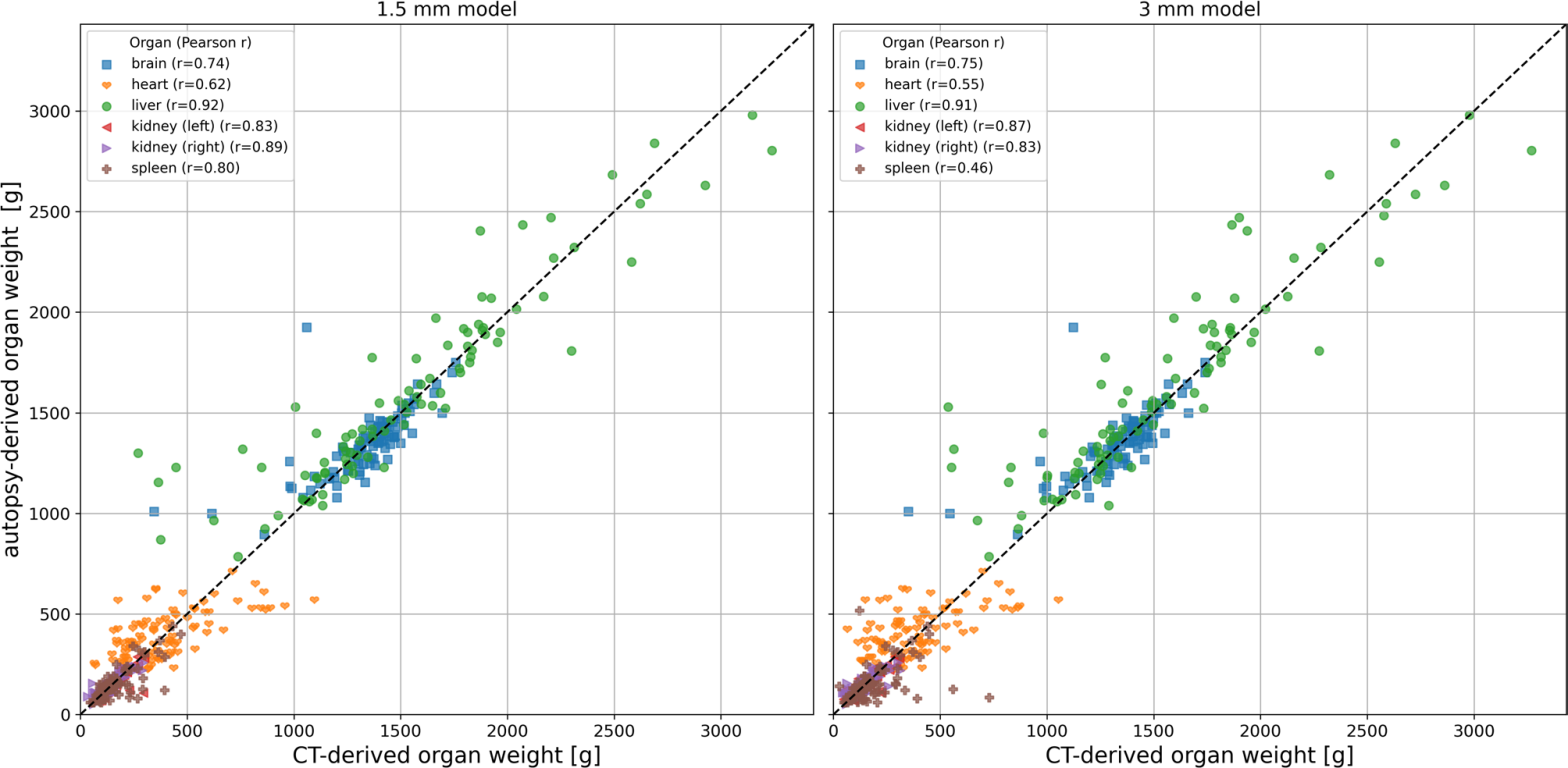
Comparison of CT- and autopsy-derived organ weights for the 1.5 mm model (left) and the 3 mm model (right), referring to the AI segmentation resolution. Each point represents one organ measurement from a single individual. The dashed diagonal line represents the line of identity, indicating perfect agreement between both measurement methods. Deviations from this line indicate overestimation or underestimation of organ weight by CT-based estimation. Pearson correlation coefficients are shown in the legends of each panel (see also Supplementary Figure S2 for Bland-Altman analysis).

**Fig. 3:**
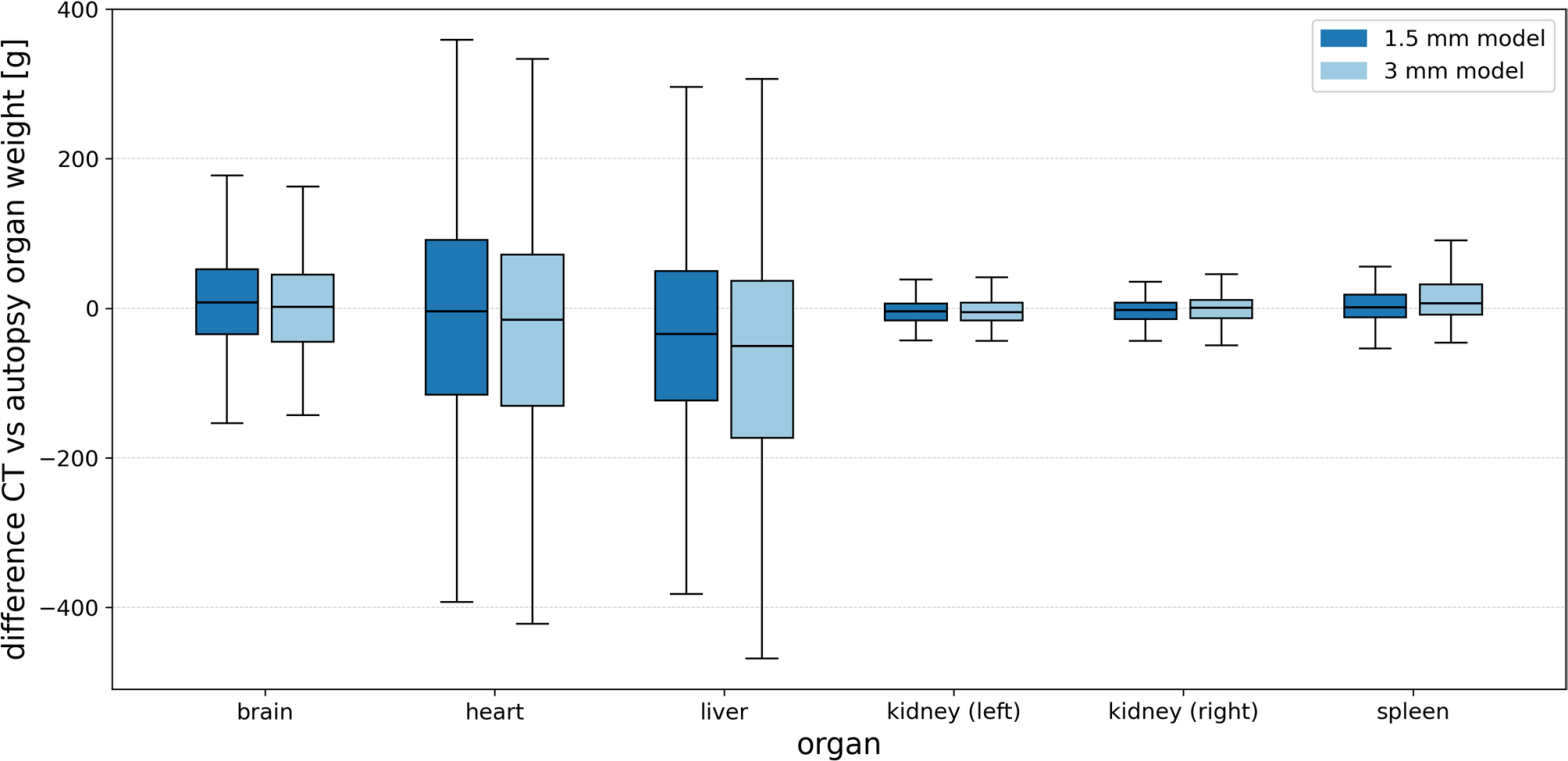
Boxplots illustrating the distribution of differences between CT- and autopsy-derived organ weights for the 1.5 mm and 3 mm models. Negative values indicate underestimation of organ weight by CT-derived measurements.

Regarding individual organs, CT-derived organ weight estimation was most accurate for the brain, followed by the liver, with good agreement also observed for the kidneys and spleen. The largest deviations occurred for the heart. Overall, both the 1.5 mm and 3 mm models showed comparable performance, with small organ-dependent differences in bias (range approximately − 20 to + 34 g). Bland-Altman analysis (Supplementary Figure S2) confirmed wide limits of agreement, particularly for the liver. Nevertheless, statistically significant differences between models were observed for selected organs, including the brain, heart, liver, and spleen (*p* < 0.001). No significant differences were found for the left kidney (*p* = 0.35), and the right kidney showed only a minor effect (*p* = 0.0049). These patterns are illustrated in Fig. 2, which compares individual CT-derived and forensic autopsy measurements. Figure 3 summarizes the distribution of differences across organs for both models. The corresponding inter-model comparison in successfully segmented cases is summarized in Table 2.

**Table 2:** Segmentation performance and inter-model differences in successfully segmented cases (i.e., cases without segmentation failures, which are described in Table 4). Although the cohort comprised 100 autopsy cases, organ-specific reference weights were unavailable in some cases, resulting in reduced sample sizes for brain and kidney analyses.

Parameter	Brain	Heart	Liver	Kidney (left)	Kidney (right)	Spleen
Autopsy-derived organ weights [n]	95	100	100	99	98	100
1.5 mm model [n]	88	92	92	95	94	95
3 mm model [n]	89	93	91	96	93	99
Differences between models in successfully segmented cases [g]						
abs. mean ± SD	14 ± 14	25 ± 22	60 ± 88	7 ± 11	8 ± 14	25 ± 61
abs. median	11	19	28	3	5	7
min	0	1	0	0	0	0
max	70	100	470	72	117	499

**Fig. 4:**
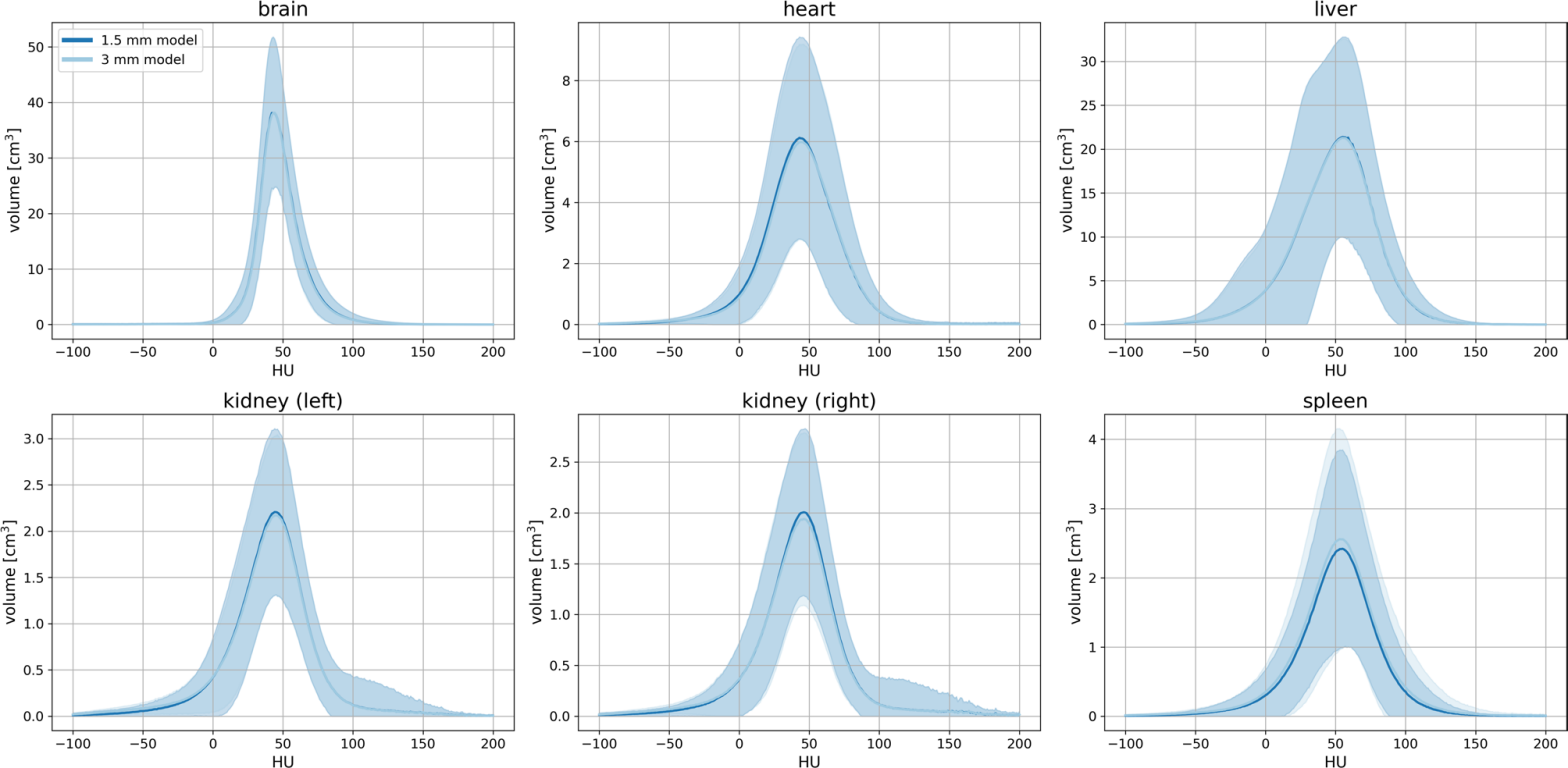
Voxel volume distribution across Hounsfield units (HU) for postmortem CT-derived organ segmentations using 1.5 mm and 3 mm model resolutions. For clarity, the HU range is restricted to -100 to 200 HU. Solid lines represent the mean voxel volume per HU, shaded areas indicate the standard deviation.

**Fig. 5:**
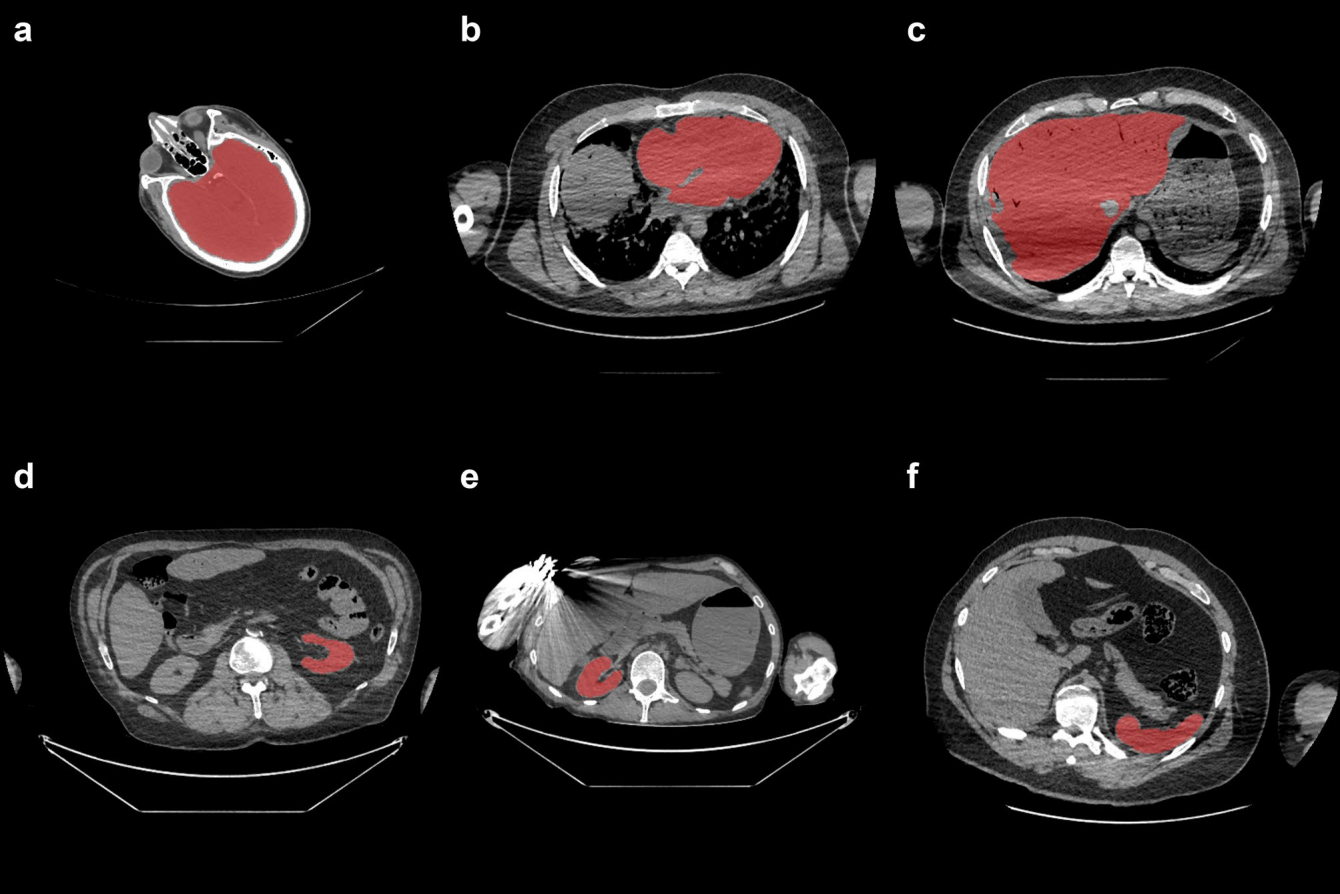
Representative examples of successful AI-based organ segmentations in postmortem CT data. For visualization purposes, single representative CT slices from 3D segmentations are shown for (**a**) brain, (**b**) heart, (**c**) liver, (**d**) left kidney, (**e**) right kidney, and (**f**) spleen. These examples demonstrate accurate anatomical delineation in cases without major decomposition, trauma, or imaging artifacts.

**Fig. 6:**
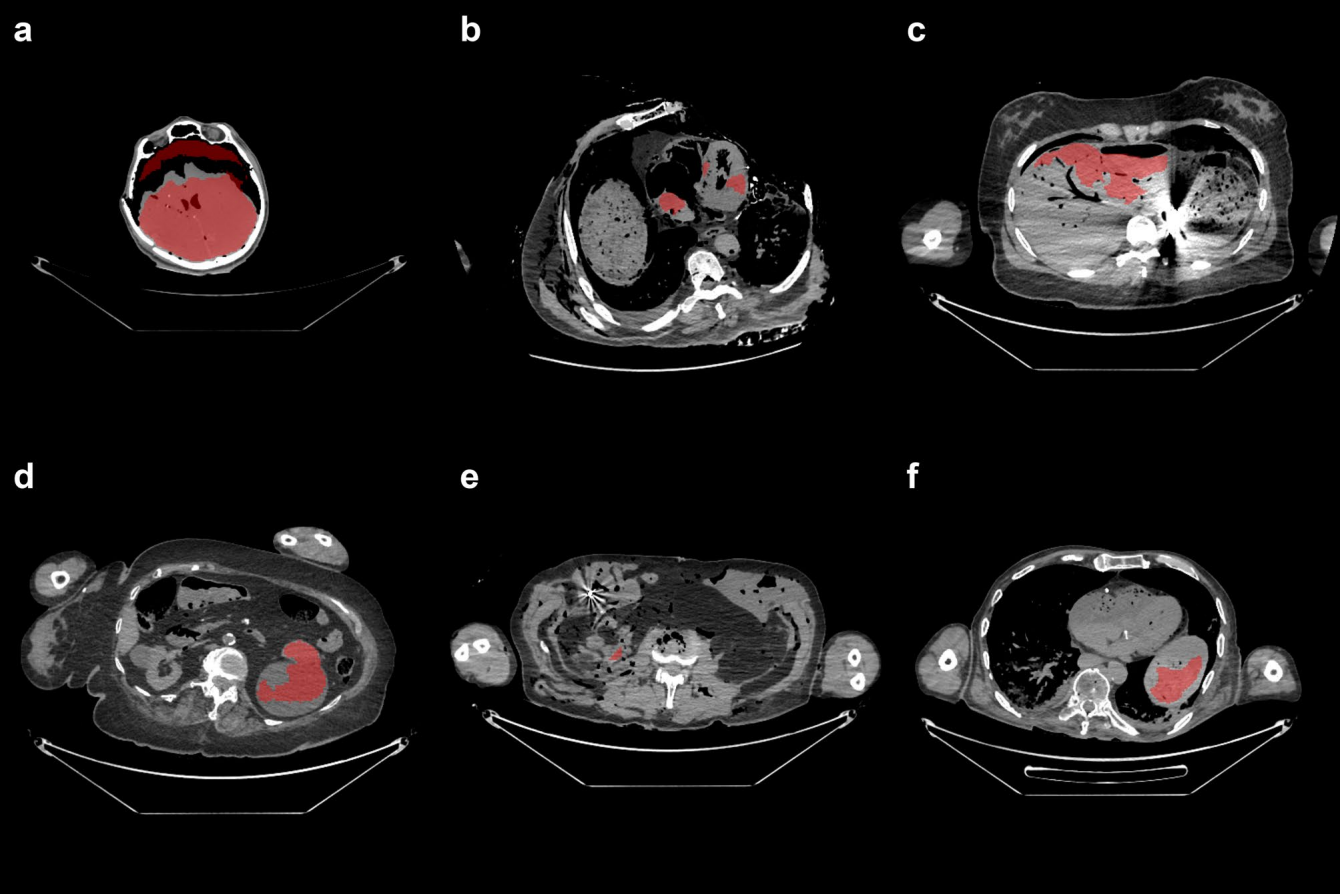
Representative examples of failed or inaccurate AI-based organ segmentations in postmortem CT data. For visualization purposes, single representative CT slices from 3D segmentations are shown for (**a**) brain, (**b**) heart, (**c**) liver, (**d**) left kidney, (**e**) right kidney, and (**f**) spleen. Segmentation errors were observed in cases with postmortem decomposition (b, c), traumatic injury (e), thermal injury, and other postmortem or pathological changes affecting tissue morphology. Such conditions may impair organ delineation and lead to inaccurate CT-derived organ weight estimation.

Analysis of HU-based volumes (see Fig. 4) revealed substantial interindividual variability, which may contribute to the observed variability between CT- and autopsy-derived organ weights. Despite these potential confounders, AI-based segmentation successfully extracted the majority of target organs in postmortem CT scans (see Fig. 5). Segmentation failures were limited to a few cases per organ and were primarily associated with conditions affecting anatomical integrity and tissue preservation, including trauma, burns, and decomposition, with decomposition being the most frequent cause of failed segmentation (see Fig. 6). In addition, some failures resulted from partial organ coverage, for example when the brain was not fully captured because the whole-body scan started at mid-head. Overall, both models performed similarly, but segmentation sometimes succeeded in one model and failed in the other. In total, the 1.5 mm model failed in 13 cases where the 3 mm model succeeded. Conversely, the 3 mm model failed in 8 cases where the 1.5 mm model was successful. In these cases, estimates from the alternative model showed high variability, ranging from very good to very poor agreement, suggesting that postmortem-related alterations could not be reliably compensated for.

A detailed subgroup analysis of CT-derived organ weight estimation and segmentation failures stratified by postmortem condition and PMI is provided in Tables 3 and 4 as well as Supplementary Tables S3 and S4. The largest CT–autopsy discrepancies were observed in cases with decomposition, particularly affecting the liver, heart, and spleen. Thermal injury was also associated with increased variability, especially for the heart, whereas kidney measurements remained comparatively stable across subgroups. Segmentation failures followed a similar pattern, occurring most frequently in decomposed cases and less commonly in trauma-associated subgroups. A single case with extended PMI (> 10 days) showed markedly higher deviations and reduced segmentation robustness, while no consistent trend across PMI strata was observed overall.

**Table 3:** Median absolute differences between CT- and autopsy-derived organ weights for the 1.5 mm model in successfully segmented cases stratified by postmortem and clinical characteristics. Interquartile ranges (Q1–Q3) and corresponding results for the 3 mm model are provided in the Supplementary Table S3. Organ-specific effective sample sizes (n) are reported in Table 2.

Absolute median differences between CT- and autopsy-derived organ weights [g]						
Parameter	Brain	Heart	Liver	Kidney (left)	Kidney (right)	Spleen
All	44	96	73	11	11	14
Decomposition	51	185	395	8	8	31
Thermal injury	121	171	72	9	15	21
Polytrauma	30	95	99	5	9	9
Exsanguination	40	85	74	11	10	13
Cerebral edema	35	91	82	13	14	14
Pulmonary edema	39	95	121	13	10	15
PMI: 0 days	48	116	50	11	8	14
PMI: <3 days	24	96	67	9	11	12
PMI: <5 days	84	91	75	11	15	18
PMI: <10 days	36	57	91	16	14	13
PMI: >10 days	103 (*n* = 1)	-	-	19 (*n* = 1)	21 (*n* = 1)	70 (*n* = 1)
PMI: unknown	51	102	77	18	9	16

**Table 4:** Subgroup analysis of segmentation failures for the 1.5 mm model stratified by postmortem condition and postmortem interval (PMI). The column n indicates the total number of cases within each subgroup; evaluable cases varied by organ due to segmentation failures and missing autopsy data (see Table 2). Segmentation failure was defined as an umbrella term comprising complete failure of mask generation (no mask) and insufficient mask quality, resulting in implausible organ weights below predefined thresholds. The corresponding results for the 3 mm model are provided in the Supplementary Table S4.

Failed segmentations (no mask / insufficient mask quality)							
Parameter	*n*	Brain	Heart	Liver	Kidney (left)	Kidney (right)	Spleen
All	100	4 / 3	2 / 6	2 / 6	3 / 1	3 / 1	3 / 2
Decomposition	10	0	0 / 3	0 / 4	0	0 / 1	0 / 2
Thermal injury	8	0	0 / 1	0	0	0	0
Polytrauma	12	0 / 1	0	0	0	0	0
Exsanguination	24	2 / 0	1 / 1	1 / 0	1 / 0	1 / 0	1 / 0
Cerebral edema	50	2 / 0	1 / 1	1 / 2	1 / 0	1 / 1	1 / 0
Pulmonary edema	28	1 / 1	0 / 1	0 / 2	1 / 0	1 / 0	1 / 1
PMI: 0 days	14	1 / 0	1 / 0	1 / 0	1 / 0	1 / 0	1 / 0
PMI: <3 days	31	2 / 0	1 / 0	1 / 0	1 / 0	1 / 0	1 / 0
PMI: <5 days	13	1 / 1	0	0	0	0	0
PMI: <10 days	8	0 / 1	0 / 1	0 / 1	0 / 1	0	0 / 1
PMI: >10 days	1	0	0 / 1	0 / 1	0	0	0
PMI: unknown	33	0 / 1	0 / 4	0 / 4	1 / 0	1 / 1	1 / 1

Systematic variation of tissue density values resulted in only minor changes in CT-derived organ weight estimates (see Table 5; Supplementary Figure S5). The largest improvements were observed for the kidneys and spleen, whereas brain, heart, and liver showed only minor changes across parameter settings. For the HU-dependent density model used for the evaluation, optimized scale factors were close to 1.0 for brain, heart, and liver, and higher for both kidneys and the spleen.

**Table 5:** Sensitivity analysis of CT-derived organ weight estimation across density assumptions and HU scaling. Values are median absolute error [Q1–Q3] relative to autopsy-derived organ weights. Additional details on the underlying density and scaling variations are provided in Supplementary Figure S5. Organ-specific effective sample sizes (n) used for the statistical analyses are reported in Table 2.

Absolute median differences [Q1–Q3] between CT- and autopsy-derived organ weights [g]						
Parameter	Brain	Heart	Liver	Kidney (left)	Kidney (right)	Spleen
1.5 mm model						
Literature-based fixed density [g]	52 [27, 83]	98 [63, 171]	72 [41, 160]	22 [13, 34]	23 [13, 35]	18 [7, 34]
Optimized fixed density [g]	45 [28, 90]	97 [63, 170]	72 [41, 160]	10 [6, 20]	11 [6, 21]	14 [5, 37]
HU-based densities (factor 1.0) [g]	45 [24, 88]	101 [62, 168]	73 [39, 172]	22 [15, 37]	24 [14, 36]	18 [7, 35]
Optimized HU-based densities [g]	44 [24, 91]	96 [65, 172]	73 [39, 172]	11 [5, 22]	11 [5, 21]	14 [5, 37]
Optimized scale factor	1.01	1.03	1.00	1.14	1.17	1.07
3 mm model						
Literature-based fixed density [g]	60 [24, 91]	106 [65, 183]	83 [40, 192]	21 [12, 34]	25 [13, 35]	21 [7, 51]
Optimized fixed density [g]	48 [27, 92]	103 [66, 176]	76 [46, 202]	11 [6, 22]	13 [7, 25]	16 [7, 58]
HU-based densities (factor 1.0) [g]	54 [23, 92]	105 [61, 174]	78 [46, 187]	22 [14, 37]	26 [14, 37]	21 [8, 49]
optimized HU-based densities [g]	45 [29, 90]	103 [64, 175]	70 [48, 208]	11 [6, 24]	12 [7, 25]	16 [8, 59]
Optimized scale factor	1.01	1.01	0.99	1.14	1.20	1.09

## Discussion

Organ weight estimation from postmortem CT using AI-based segmentation demonstrates good agreement with conventional autopsy measurements. Overall differences between CT- and autopsy-derived organ weights were relatively small. Performance was comparable between the 1.5 mm and 3 mm models, with organ-specific variations in accuracy. These findings further support the use of postmortem CT in forensic practice. They also indicate that CT-based organ weight estimation may provide additional quantitative information, especially when a conventional autopsy is not performed.

Most previous studies on CT-derived organ weight or volume assessment have relied on manual or semi-manual segmentation approaches rather than fully automated pipelines. For the brain, Lescot et al.^4^ compared CT-derived brain weights between 15 patients with severe traumatic brain injury and 15 matched controls. Brain weight was estimated by manual segmentation and by combining segmented volume with density estimates derived from attenuation values. Similar manual approaches have been reported for the heart by Ogawa et al.^5^, for the liver by Sonnemans et al.^6^ and Inai et al.^7^, and for liver and spleen volume estimation by Alves et al.^8^. Manual lung segmentation has also been described by Matoba et al.^12^ and experimentally by Protti et al.^13^ in porcine models. In contrast to these earlier studies, the present work evaluates a fully automated multi-organ segmentation pipeline. Simultaneous assessment of several organs is possible within a single postmortem CT examination, potentially reducing analysis time and improving reproducibility by minimizing observer-dependent variability. Table 6 summarizes the studies that could be directly compared with the present work, i.e., those reporting autopsy-based reference data and sufficient numerical information for extraction or approximation of performance metrics. Direct comparison is limited by differences in study design, cohort characteristics, PMI, imaging protocols, and reported performance metrics. Furthermore, Lescot et al.^4^ and Alves et al.^8^ investigated living subjects rather than postmortem cases, and some performance measures in previous studies had to be approximated from published figures due to incomplete numerical reporting. For heart weight estimation, relatively large prediction errors were observed in the available data, including both Ogawa et al.^5^ and the present study, suggesting that accurate CT-based assessment of heart weight remains challenging. Although the present study showed slightly lower median errors, this difference should be interpreted cautiously. For liver weight estimation, the fully automated approach achieved accuracy comparable to previously reported manual segmentation methods^6,7^. These findings suggest that automated multi-organ segmentation can provide performance similar to manual approaches while substantially reducing the required workload.

**Table 6:** Comparison of published studies on CT-derived organ weight or volume estimation with the present study. For the study reporting volume only, values were converted to weight using a fixed density selected to achieve the best overall agreement. Values are median absolute error [Q1–Q3] relative to autopsy-derived organ weights and reported accuracy metrics (percentage of cases within ± 5% and ± 10% error). Values marked as approximated were obtained by digital extraction from published figures and should therefore be considered estimates. Direct comparisons between studies are limited by differences in PMI, imaging protocols, cohort characteristics, and outcome definitions. Therefore, the reported inter-study differences should be interpreted as indicative rather than strictly quantitative comparisons.

Study	Cohort	Organ	abs. median [Q1–Q3] [g]	Error ≤ ± % / ±1% [%]	PMI [days]	Notes
Ogawa et al.^5^	33	Heart	120 [80, 155]	6 / 13	0	Approximated from figure
This study (1.5 mm model)	13	Heart	116 [25, 198]	15 / 31	0	
	92	Heart	96 [65, 172]	9 / 16	various	
Sonnemans et al.^6^	39	Liver	105 [51, 156]	49 / 79	0	Fixed density 1.01 g/cm^3^
Inai et al.^7^	30	Liver	65 [40, 98]	43 / 77	0	Approximated from figure
This study (1.5 mm model)	13	Liver	50 [28, 167]	54 / 77	0	
	92	Liver	73 [39, 172]	47 / 76	various	

The level of agreement differed notably between organs. The brain and liver showed the best overall performance, while the heart remained the most challenging organ. Among the investigated organs, the heart is the only hollow organ, whereas the others are parenchymatous. Its cavernous nature makes it vulnerable to gas accumulation even in early decomposition, which leads to segmentation difficulties and lower estimated organ weights. In addition, blood within the heart affects weight estimation (see Supplementary Figure S6). Usually, the heart contains blood after death, up to approximately 550 ml^5^, at least until decomposition begins or massive exsanguination occurs. In conventional autopsy, organ weight is measured after the heart has been drained of blood. It was therefore expected that blood content in cases with short PMI and no signs of decomposition would lead to higher estimated organ weights. However, the expected pattern was not observed uniformly, with underestimation of heart weight also occurring in non-exsanguinated cases, possibly due to segmentation difficulties in blood-filled cardiac chambers. A possible explanation for these diverging tendencies is the condition of the blood. Depending on individual circumstances, blood may range from fluid to different stages of coagulation, which affects segmentation. The organ structure itself and individual anatomical differences further complicate segmentation. Additionally, postmortem myocardial relaxation alters cardiac configuration. This may cause difficulties for a segmentation tool trained on living humans with a beating heart. The difference between living individuals and postmortem scans is, for the heart, much more pronounced than for static organs such as the liver. Thus, the wide spread observed for the heart likely reflects a combination of segmentation difficulty and postmortem-related changes rather than a single systematic bias. Further studies, especially training TotalSegmentator on postmortem cases, are needed to improve heart weight estimation and better understand these challenges. By contrast, the kidneys were estimated with relatively high consistency, which may reflect their compact structure and stable CT delineation. Heimer et al.^14^ showed that blood loss can reduce organ weight and lung attenuation, while spleen and kidney attenuation remain largely stable, and liver attenuation may even increase, possibly due to hepatic glycogen content. These organ-specific effects of hemorrhage may partly explain residual deviations in CT-derived organ weight estimation, for example in cases of fatal bleeding due to stab wounds.

The present findings indicate that variations in CT acquisition parameters and HU-based density modeling contribute only marginally to overall measurement uncertainty. This includes differences in slice thickness and density assumptions. The kidney-specific scale factors suggest a modest systematic underestimation of renal mass. Because a similar pattern was also seen with the fixed-density approach (see Supplementary Figure S5), the deviation is unlikely to be driven primarily by the HU-to-density transformation alone. Instead, it may reflect a small systematic undersegmentation at organ margins or partial-volume effects, which would affect both modelling strategies in a similar way. This stability is consistent with the fact that TotalSegmentator was trained on heterogeneous routine clinical CT data from living patients across a wide range of scanners and acquisition settings^9^. This likely contributes to its robustness against technical variability. In contrast, the primary source of uncertainty appears to be segmentation quality. Postmortem structural alterations may impair organ delineation or lead to segmentation failure, which in turn accounts for the largest deviations between CT- and autopsy-derived organ weights. This is supported by the subgroup analyses, which did not demonstrate a consistent association between PMI and measurement deviations, suggesting that the overall structural condition of the body is more relevant than time since death alone. Accordingly, postmortem decomposition represents a major challenge for robust automated segmentation. Postmortem-specific training data and algorithmic adaptation to decomposition-related changes may therefore further improve performance in forensic applications.

Beyond organ weight estimation itself, the present findings may be relevant for future computer vision-based identification^15–19^ workflows. However, this application was not directly evaluated in the present study. In principle, automatically derived organ weights could be considered as additional constraints when querying antemortem reference databases, alongside variables such as sex^20,21^, age^15,22^, or estimated body weight^3,23–25^. In this context, organ-specific measurements would not serve as identifiers, but could help restrict the pool of candidate reference cases. Given potential variation due to disease, treatment, physiological changes, or interscan differences, organ weights should be interpreted as probabilistic and non-specific parameters. Further studies are required to assess any practical utility of this concept. Furthermore, a reliable method of automated organ weight estimation may also have potential clinical applications. One example is preoperative assessment in liver transplantation^26,27^, where organ size is an important parameter for donor-recipient matching. In addition, it may support large-scale research applications, such as epidemiological studies of organ hypertrophy or population-based morphological analyses^28,29^. The present study is methodological, and routine application remains future-oriented. In a potential workflow, postmortem CT data could be automatically processed after DICOM export from the scanner or PACS using a standalone pipeline for segmentation and organ weight estimation. Before such use, external validation across different scanners, protocols, and forensic populations would be required, together with quality assurance procedures to detect segmentation failures, particularly in cases with postmortem changes. Computational requirements are modest and allow processing on standard workstation hardware. User training would be necessary to ensure appropriate interpretation of results, particularly in cases with postmortem changes, segmentation uncertainty, or atypical anatomy.

Despite these promising results, several limitations should be considered when interpreting the findings. First, autopsy measurements, although regarded as the reference standard, are themselves subject to procedural variability related to dissection technique, fluid loss, and organ handling. Second, this was a retrospective single-center study. Only cases with available postmortem CT examinations and documented autopsy organ weights were included, introducing a potential selection bias and limiting generalizability. Third, several subgroup analyses were additionally based on small sample sizes and should therefore be interpreted as exploratory. Furthermore, the scaling parameters were optimized and evaluated within the same cohort. Therefore, the reported performance may be slightly optimistic and should be validated in an independent external dataset. Finally, the segmentation algorithm was originally trained on antemortem clinical CT data. This introduces a domain shift when the model is applied to postmortem imaging, particularly in cases with decomposition, trauma, or severe anatomical alteration. While performance was generally good, postmortem-specific training datasets may further improve robustness in challenging forensic cases. In addition, future research may explore the extension of this volumetric approach to other imaging modalities such as magnetic resonance imaging (MRI); however, susceptibility artifacts in MRI^30^ may influence the robustness of quantitative measurements and would need to be systematically addressed.

In conclusion, AI-based organ segmentation allows feasible estimation of organ weights from postmortem CT and shows overall good agreement with conventional autopsy measurements. The best performance was observed for the brain, liver, kidneys, and spleen, whereas the heart remained the most challenging organ. Although segmentation failures occurred in a minority of cases, they were mainly associated with decomposition, trauma, burns, or incomplete organ coverage. Future studies should focus on larger multicenter cohorts, postmortem-specific AI training datasets, and more advanced correction of tissue density changes after death. Such developments may further improve the reliability of CT-derived organ weight estimation. They may also support its integration into forensic routine practice, epidemiological studies, and potentially also antemortem identification workflows.

## Supplementary Information

Below is the link to the electronic supplementary material.

Supplementary Material

## Data Availability

The datasets analyzed during the current study are not publicly available due to privacy concerns. However, all relevant data supporting the findings of this study are included in the article. For further inquiries or data requests, please contact the corresponding author.
