# Supplementary material for: AI-based organ weight estimation from postmortem computed tomography
Source: Sci Rep. 2026 Jul 14;16:22032. doi: 10.1038/s41598-026-62085-2 (PMC13365199; doi:10.1038/s41598-026-62085-2)
Supplement: Supplementary file 1 — Supplementary Material [file 41598_2026_62085_MOESM1_ESM.pdf]

AI-based organ weight estimation from postmortem Computed Tomography

Marc Windgassen[1], Andreas Heinrich[2]

[1]Institute of Forensic Medicine, Jena University Hospital –
Friedrich Schiller University, Am Klinikum 1, 07747 Jena, Germany

[2]Department of Radiology, Jena University Hospital –
Friedrich Schiller University, Am Klinikum 1, 07747 Jena, Germany

Table S1 Summary statistics of CT- and autopsy-derived organ weights and their case-wise differences. Values are reported as mean ± standard deviation (SD) or median [Q1–Q3]. Differences were calculated as CT- minus autopsy-derived organ weight. Percentage values represent the median absolute percentage deviation relative to autopsy-derived organ weights.

**parameter**	**brain**	**heart**	**liver**	**kidney (left)**	**kidney (right)**	**spleen**
autopsy-derived organ weights [g]	1355 ± 167	402 ± 117	1596 ± 510	150 ± 47	142 ± 43	152 ± 85
1.5 mm model CT-derived organ weights [g]	1313 ± 266	403 ± 207	1548 ± 583	147 ± 52	137 ± 52	163 ± 88
3 mm model CT-derived organ weights [g]	1305 ± 260	387 ± 198	1552 ± 554	146 ± 53	141 ± 56	180 ± 111
1.5 mm model - differences between CT- and autopsy-derived organ weights [g]						
mean ± SD	-8 ± 146	-1 ± 164	-74 ± 233	-2 ± 29	-4 ± 23	13 ± 53
abs. mean ± SD	79 ± 123	129 ± 100	152 ± 191	17 ± 23	16 ± 17	31 ± 45
median [Q1–Q3]	8 [-34, 52]	-4 [-115, 92]	-34 [-123, 50]	-4 [-16, 6]	-2 [-15, 8]	2 [-12, 18]
abs. median [Q1–Q3]	44 [24, 91]	96 [65, 172]	73 [39, 172]	11 [5, 22]	11 [5, 21]	14 [5, 37]
percentage	4	29	5	7	8	12
3 mm model - differences between CT- and autopsy-derived organ weights [g]						
mean ± SD	-18 ± 142	-24 ± 166	-91 ± 231	-4 ± 26	0 ± 30	26 ± 104
abs. mean ± SD	80 ± 119	133 ± 101	162 ± 188	18 ± 20	20 ± 22	51 ± 95
median [Q1–Q3]	2 [-45, 45]	-15 [-130, 72]	-50 [-173, 37]	-5 [-16, 8]	1 [-13, 11]	7 [-8, 32]
abs. median [Q1–Q3]	45 [29, 90]	103 [64, 175]	70 [48, 208]	11 [6, 24]	12 [7, 25]	16 [8, 59]
percentage	3	29	5	8	10	13

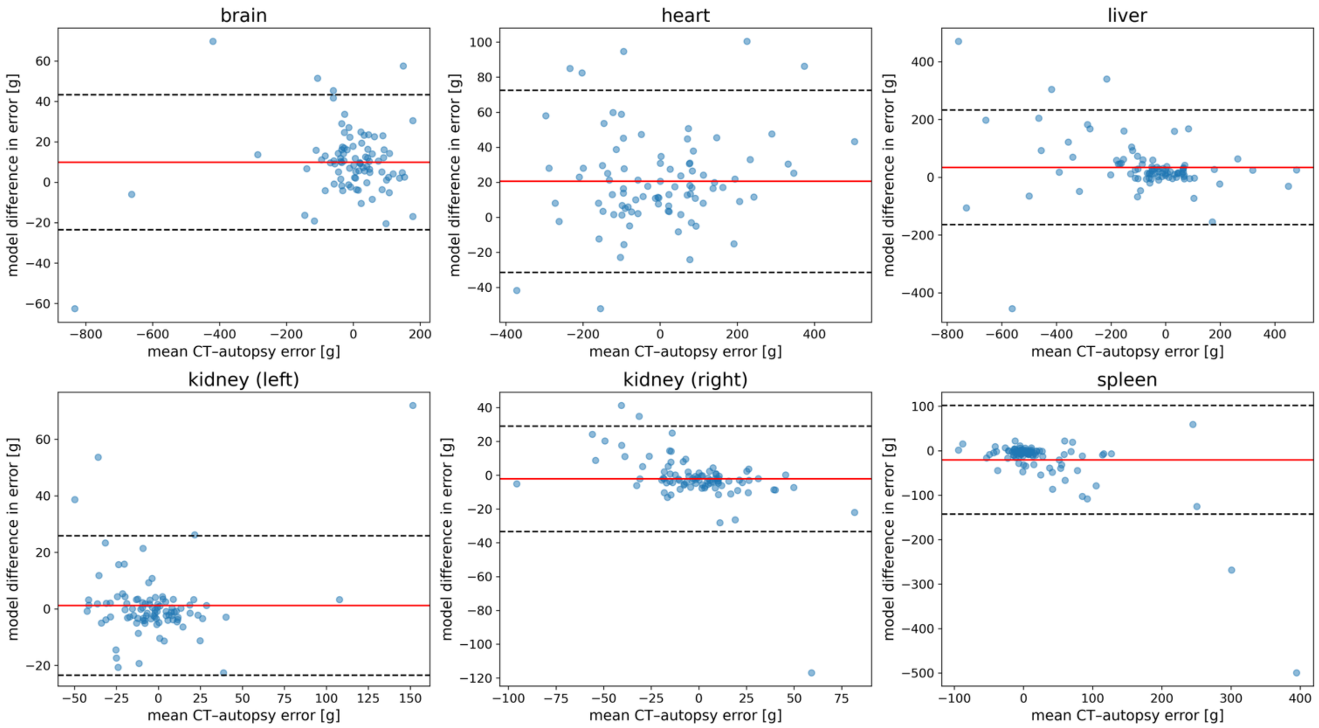

Figure S2 Bland-Altman plots comparing CT-autopsy differences between models. For each subject, the difference was defined as the CT-autopsy error of the 1.5 mm model minus that of the 3 mm model.

Table S3 Median absolute differences between CT- and autopsy-derived organ weights in successfully segmented cases stratified by postmortem and clinical characteristics.

absolute median differences [Q1–Q3] between CT- and autopsy-derived organ weights [g]

parameter	brain	heart	liver	kidney (left)	kidney (right)	spleen
			1.5 mm model			
all	44 [24, 91]	96 [65, 172]	73 [39, 172]	11 [5, 22]	11 [5, 21]	14 [5, 37]
decomposition	51 [38, 86]	185 [92, 220]	395 [128, 543]	8 [4, 16]	8 [5, 10]	31 [22, 61]
thermal injury	121 [74, 139]	171 [93, 237]	72 [66, 283]	9 [4, 18]	15 [4, 24]	21 [10, 48]
polytrauma	30 [21, 38]	95 [86, 132]	99 [30, 126]	5 [3, 10]	9 [5, 22]	9 [4, 15]
exsanguination	40 [19, 80]	85 [73, 123]	74 [37, 131]	11 [7, 15]	10 [5, 19]	13 [4, 32]
cerebral edema	35 [19, 76]	91 [63, 174]	82 [54, 153]	13 [7, 27]	14 [5, 29]	14 [4, 34]
pulmonary edema	39 [33, 89]	95 [72, 249]	121 [67, 363]	13 [6, 24]	10 [4, 23]	15 [7, 25]
PMI: 0 days	48 [23, 80]	116 [25, 198]	50 [28, 167]	11 [5, 19]	8 [2, 12]	14 [4, 46]
PMI: <3 days	24 [10, 63]	96 [72, 123]	67 [36, 144]	9 [5, 15]	11 [4, 20]	12 [6, 51]
PMI: <5 days	84 [34, 120]	91 [54, 151]	75 [68, 139]	11 [7, 30]	15 [7, 23]	18 [4, 27]
PMI: <10 days	36 [28, 95]	57 [43, 185]	91 [64, 105]	16 [8, 20]	14 [7, 29]	13 [8, 37]
PMI: >10 days	103 (n=1)	-	-	19 (n=1)	21 (n=1)	70 (n=1)
PMI: unknown	51 [36, 88]	102 [85, 175]	77 [39, 305]	18 [5, 30]	9 [5, 21]	16 [8, 27]
			3 mm model			
all	45 [29, 90]	103 [64, 175]	70 [48, 208]	11 [6, 24]	12 [7, 25]	16 [8, 59]
decomposition	80 [53, 83]	201 [78, 236]	325 [187, 502]	10 [8, 18]	12 [7, 16]	46 [14, 86]
thermal injury	117 [52, 137]	165 [95, 191]	70 [59, 242]	9 [2, 17]	18 [7, 27]	20 [9, 64]
polytrauma	40 [22, 45]	129 [87, 142]	64 [37, 149]	7 [2, 12]	17 [8, 27]	11 [4, 26]
exsanguination	43 [27, 83]	103 [67, 149]	90 [58, 155]	11 [7, 16]	10 [5, 17]	15 [8, 62]
cerebral edema	40 [25, 74]	95 [61, 199]	80 [49, 180]	14 [7, 28]	17 [8, 30]	16 [8, 44]
pulmonary edema	42 [19, 84]	108 [57, 249]	104 [49, 394]	13 [7, 29]	11 [5, 26]	19 [8, 58]
PMI: 0 days	39 [29, 72]	116 [35, 221]	62 [38, 203]	11 [5, 18]	12 [6, 23]	11 [6, 81]
PMI: <3 days	37 [17, 58]	96 [68, 130]	80 [47, 158]	9 [6, 19]	16 [7, 30]	15 [10, 41]
PMI: <5 days	77 [43, 118]	97 [59, 144]	77 [67, 188]	11 [9, 18]	9 [5, 25]	22 [15, 45]
PMI: <10 days	41 [30, 90]	59 [23, 183]	65 [54, 159]	9 [8, 21]	20 [11, 26]	24 [12, 55]
PMI: >10 days	119 (n=1)	422 (n=1)	-	24 (n=1)	32 (n=1)	48 (n=1)
PMI: unknown	53 [36, 96]	134 [82, 188]	106 [43, 284]	16 [7, 29]	12 [8, 23]	14 [6, 63]

Table S4 Subgroup analysis of segmentation failures for the 3 mm model stratified by postmortem condition and postmortem interval (PMI). The final row summarizes cases in which segmentation failure occurred in both the 1.5 mm and 3 mm models. The column n indicates the total number of cases within each subgroup; evaluable cases varied by organ due to segmentation failures and availability of autopsy data (see Table 2). Segmentation failure was defined as an umbrella term comprising complete failure of mask generation (no mask) and insufficient mask quality, resulting in implausible organ weights below predefined thresholds.

failed segmentations (no mask / insufficient mask quality)

parameter	n	brain	heart	liver	kidney (left)	kidney (right)	spleen
all	100	3 / 3	1 / 6	2 / 7	3 / 0	2 / 3	1 / 0
decomposition	10	0	0 / 4	1 / 5	0	1 / 0	0
thermal injury	8	0	0	0 / 1	0	0	0
polytrauma	12	0 / 1	0	0	0	0	0
exsanguination	24	1 / 0	0 / 3	0 / 1	0	0 / 1	0
cerebral edema	50	1 / 0	0 / 1	0 / 2	0	1 / 2	0
pulmonary edema	28	1 / 1	0	0 / 2	1 / 0	0 / 2	0
PMI: 0 days	14	0	0	0	0	0	0
PMI: <3 days	31	2 / 0	1 / 0	1 / 0	1 / 0	1 / 0	1 / 0
PMI: <5 days	13	1 / 1	0 / 1	0	0	0 / 1	0
PMI: <10 days	8	0 / 1	0 / 1	0 / 1	1 / 0	0	0
PMI: >10 days	1	0	0	1 / 0	0	0	0
PMI: unknown	33	0 / 1	0 / 4	0 / 6	1 / 0	1 / 2	0
1.5 mm and 3 mm models failed	100	3 / 3	1 / 2	1 / 6	2 / 0	1 / 1	1 / 0

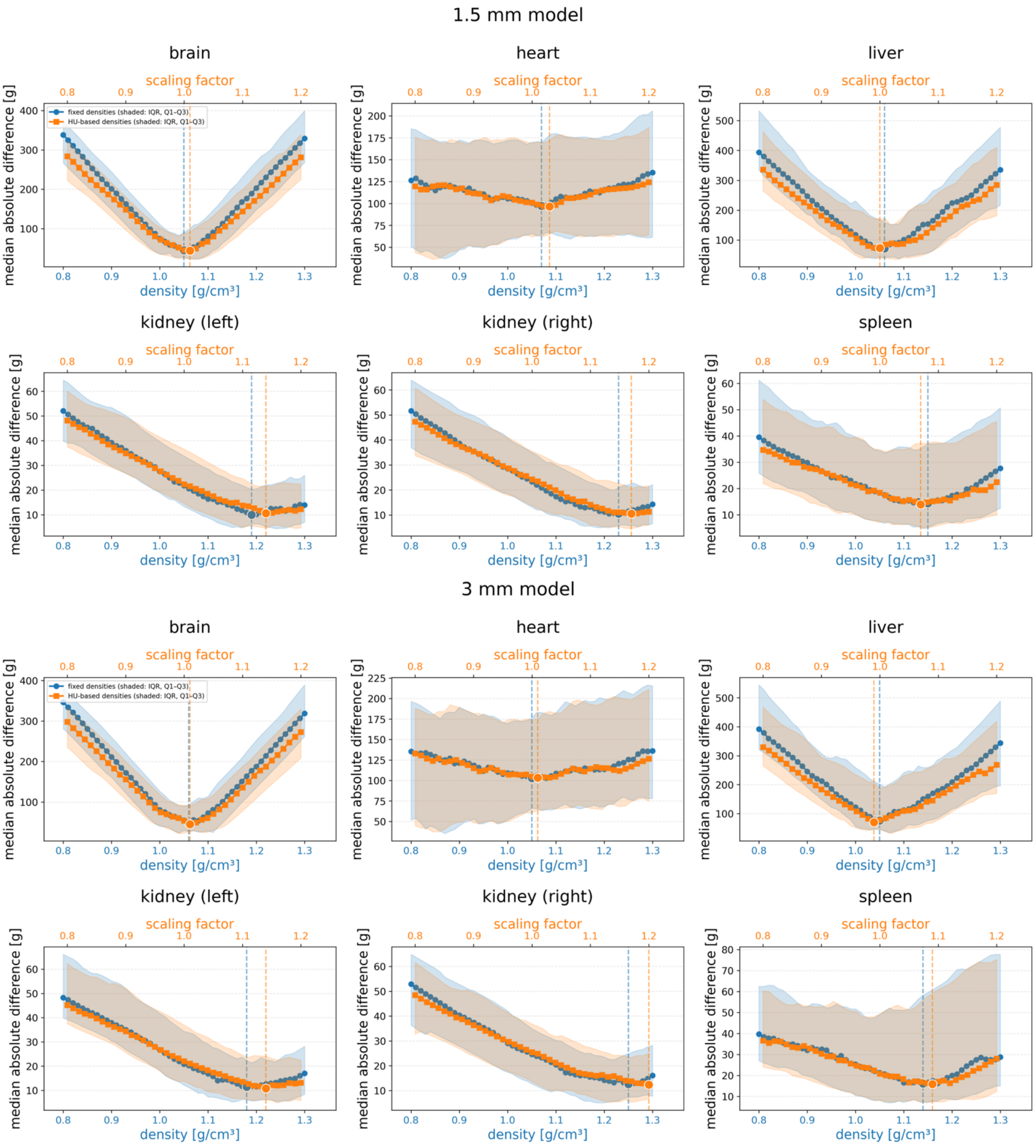

Figure S5 Sensitivity analysis of CT-derived organ weight estimation across density assumptions and HU scaling factors. The upper row shows results obtained with the 1.5 mm model, whereas the lower row shows results obtained with the 3 mm model. Solid lines represent median absolute differences between CT- and autopsy-derived organ weights. Shaded regions indicate the interquartile range (Q1–Q3). Markers and dashed vertical lines denote the parameter setting yielding the lowest median absolute difference.

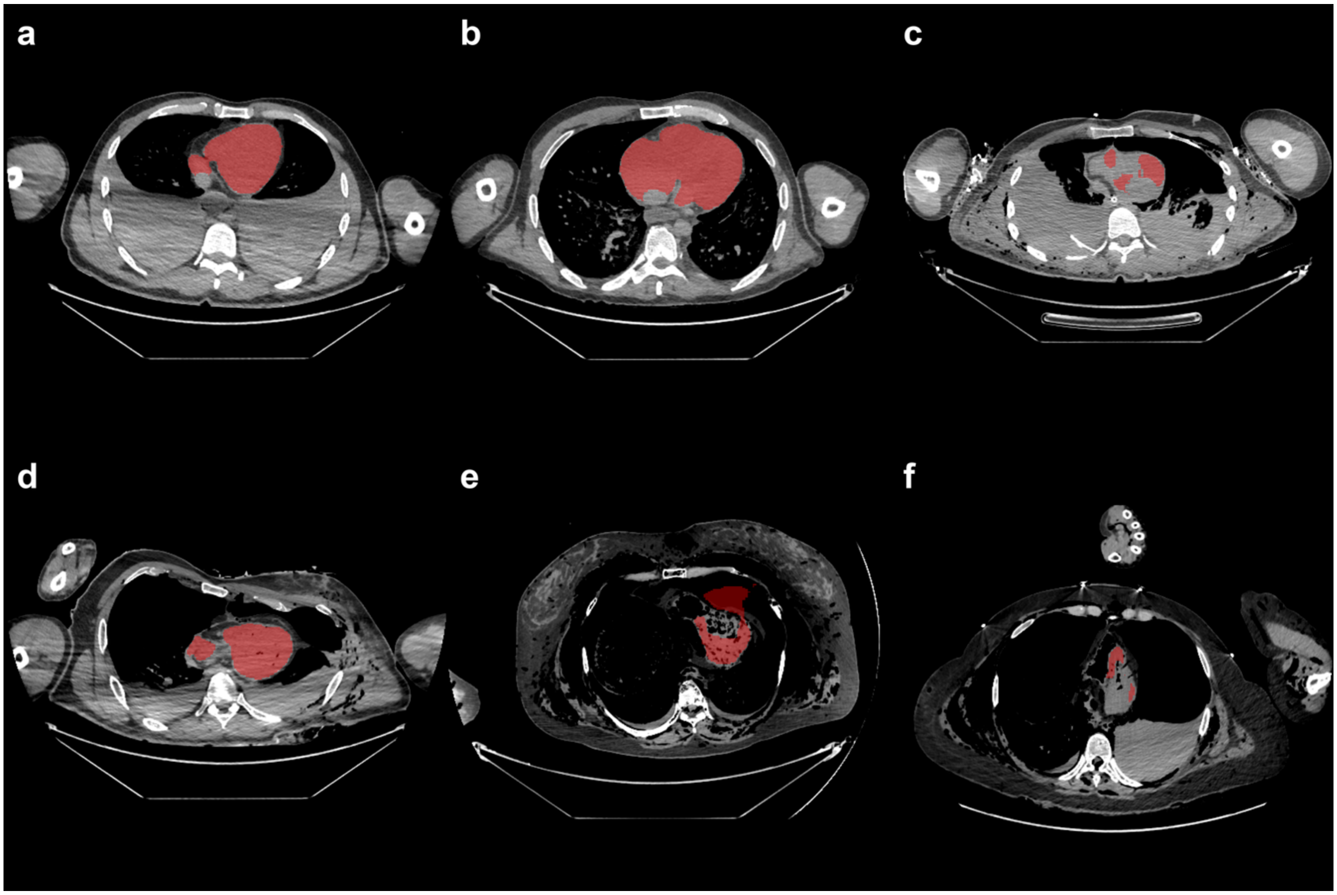

Figure S6 Examples of heart segmentations in cases with blood-filled hearts (a-c) and decomposition-related changes (d-f). Blood within the heart may result in higher estimated heart weights than autopsy weights (a). This effect may be more pronounced in larger hearts (b). Conversely, postmortem changes may lead to segmentation failure and substantial underestimation of heart weight (c). In cases of decomposition, segmentation may show only minor deviations (d), but inaccurate segmentations may also result in either overestimation (e) or underestimation (f) of heart weight. For visualization purposes, single representative CT slices from the three-dimensional segmentations are shown.
